# Disseminated *Talaromyces marneffei* Infection in a Non-HIV Infant With a Homozygous Private Variant of *RELB*

**DOI:** 10.3389/fcimb.2021.605589

**Published:** 2021-03-15

**Authors:** Xiaofang Ding, Han Huang, Lili Zhong, Min Chen, Fang Peng, Bing Zhang, Xinyu Cui, Xiu-An Yang

**Affiliations:** ^1^ Department of Pediatrics, First Affiliated Hospital of Hunan Normal University/Hunan Provincial People’s Hospital, Changsha, China; ^2^ Department of Biochemistry, School of Basic Medical Science, Chengde Medical University, Chengde, China

**Keywords:** *Talaromyces marneffei*, non-human immunodeficiency virus (HIV), combined immunodeficiency, *RelB*, next generation sequencing

## Abstract

**Objective:**

This study presents a relatively rare case of disseminated *Talaromyces marneffei* (*T. marneffei*) infection in an HIV-negative patient.

**Methods:**

An 8-month-old girl was hospitalized because of uncontrollable fever and cough for 6 days. Routine laboratory tests, biochemical detection, immunological tests, pathogenic examination, and imaging inspection were performed. Genetic tests of trio whole genome sequencing (Trio-WES), trio copy number sequencing (Trio-CNVseq), and Sanger sequencing were conducted to identify pathogenic variants. *In silico* analysis of the sequence alignment and structural modeling results was carried out to study the possible pathogenicity of the identified variant. Western blotting was performed to investigate the expression of the identified gene at the protein level.

**Results:**

Enhanced CT and MRI scanning demonstrated thymic dysplasia, diffuse pulmonary and liver nodules, and many balloon-like air sacs in both lungs. The white blood cell count, neutrophil count, and neutrophil ratio were normal or elevated. The patient was HIV-negative and bone marrow and blood culture showed *T. marneffei* infection. Total lymphocyte count, CD3+ T lymphocyte count, CD3+CD4+ T lymphocyte count, CD3+CD8+ T lymphocyte count, and NK cell count decreased, while the number of CD19 positive B cells increased. However, the ratio of CD3+CD4+:CD3+CD8+ T cells increased. Trio-WES identified a homozygous private variant of NM_006509: c.400_c.401insAGC/p.Lys134 delinsLysGln in *RELB* and Sanger sequencing validated the result. Structural modeling indicated that the variant may be pathogenic. Reverse transcription-polymerase chain reaction and Western blot analysis showed that the expression of RelB in the patient was lower than that in the healthy controls at mRNA and protein levels.

**Conclusion:**

This is the first report on disseminated *T. marneffei* infection in a patient with a homozygous private variant of *RELB*.

## Introduction

*Talaromyces marneffei (T. marneffei)* infection has been widely recorded as an endemic disease in Southeast Asian countries (such as Thailand, Malaysia, Myanmar, Cambodia, and Laos) and southern provinces of China (including Guangxi, Guangdong, Fujian, Hunan, Taiwan, and Hong Kong) (Vanittanakom et al., 2006). It was once considered that *T. marneffei* infection was exclusively related to acquired immunodeficiency syndrome (AIDS) induced by human immunodeficiency virus (HIV) (Chan et al., 2016). However, it was later reported in patients with other immunodeficiencies such as autoimmune diseases, solid organ or hematopoietic stem cell transplantation, hematology, and those receiving novel targeted therapies (Wang et al., 2003; Woo et al., 2005; Chan et al., 2013; Chan et al., 2015; Lei et al., 2018). *T. marneffei* infection involves multiple organ systems, such as the lungs, skin, bone marrow, digestive system, and disseminated infections (Samson et al., 2011; Qiu et al., 2019). Amphotericin B, itraconazole, voriconazole, and posaconazole are effective in the management of *T. marneffei* infection (Lei et al., 2018). Unfortunately, the mortality rate of *T. marneffei* infection is very high, especially for non-AIDS patients due to delayed diagnosis (Le et al., 2011; Lee et al., 2012; Lei et al., 2018; Qiu et al., 2019).

Primary immunodeficiency diseases (PIDs) are a group of heterogeneous inborn errors of immunity characterized by increased susceptibility to infectious diseases, autoimmunity, autoinflammatory diseases, allergy, and/or malignancy (Tangye et al., 2020). To date, more than 430 genes have been found to be associated with PIDs, including genes encoding the nuclear factor-kappa B (NF-κB) signaling pathway (Picard et al., 2018; Bousfiha et al., 2020; Chinn et al., 2020). Two NF-κB pathways have been revealed: one is a classical pathway involving RelA and c-Rel, and the other is the alternative pathway involving RelB (Sun, 2011; Zhang et al., 2017). Roifman et al. reported that 3 brothers suffered from combined immunodeficiency (CID) due to a homozygous mutation (c.C1191A/p.Y397X) in the *RELB* gene (Mericoa et al., 2015; Sharfe et al., 2015; Ovadia et al., 2017). To date, *STAT3, STAT1, TNFSF5(CD40L)*, and *IFNGR1* mutations have been found in HIV-negative patients with *T. marneffei* infection (Lee et al., 2012; Lee et al., 2014; Fan et al., 2018; Lee et al., 2019; Zhang W. et al., 2020). In this study, we present a non-HIV infant infected with *T. marneffei* and CID carrying a homozygous private variant of *RELB.*

## Materials and Methods

### Patient

An 8-month-old girl was admitted to our hospital because of a 6-day history of fever and cough. Shortness of breath lasted for 2 days. The infant is the second child of a nonconsanguineous Chinese family and has a healthy older brother. The baby was born at full term and had no history of asphyxia due to birth injury. She had a history of whooping cough and pneumonia at the ages of 4 and 6 months, respectively. There was no history of exposure to endemic water and schistosomiasis sources. Her parents and brother were clinically normal, and their family history of hereditary diseases was denied. This study was approved by the Medical Ethics Committee of Hunan Provincial People’s Hospital. Written informed consent to participate in this study was provided by the legal guardian.

### Auxiliary Examinations

Blood gas analysis was performed to measure respiratory function and acid-based balance. Blood, urine, and stool samples were examined using routine laboratory tests. Biochemical tests were conducted to study the functions of the liver and kidney. Hematological examination was carried out to investigate the conditions of hematopoiesis, blood cell differentiation, and anemia. C-reactive protein (CRP) content was tested using a Full C-Reactive Protein Quantification Test kit (Shanghai Upper Bio-Tech Pharma Co., Ltd., Shanghai, China) according to the operation guide. Enzyme linked immunosorbent assay for procalcitonin (PCT) inspection was carried out to understand the active degree of systemic inflammatory reaction using VIDAS^®^ B.R.A.H.M. S PCT™ (bioMérieux Corporate, Shanghai, China). Immune globulin was determined by turbidimetry assay using N Antiserum to Human IgA, IgG, IgM (Erlangen, Germany). Complement C3 and complement C4 were detected with turbidimetry assays with N Antiserum to Human C3c and N Antiserum to Human C4 provided by Siemens Healthineers (Erlangen, Germany). Measurement of respiratory burst activity was carried out as described by Peng et al. (2019). Immunomagnetic methods were used for Toxoplasma, rubella, cytomegalovirus, and herpes simplex virus detection. Influenza A, influenza B, respiratory syncytial virus, adenovirus, and parainfluenza types 1, 2, and 3 were tested by fluorescence immunoassay. Fluorescence polymerase chain reaction (PCR) was performed test for SARS-CoV-2 and Mycoplasma pneumoniae infection. Interferon-γ release assays were conducted on T cells using the TB-IGRA kit (cat. no. TB-0296; WANTAI BioPharm Co., Ltd., Beijing, China) to detect tubercle bacillus infection. HIV was detected by enzyme-linked immunosorbent assay. Sputum, bone marrow, and blood cultures were used to detect bacterial and fungal infections. Galactomannan enzyme immunoassay (GM-EIA) and (1–3) B-D-glucan assay (BG) were used for the diagnosis of invasive fungal infections using serum. Imaging examinations, including heart and abdominal ultrasound, chest and abdominal enhanced CT, and abdominal MRI, were conducted.

### NGS

Trio whole genome sequencing (trio-WES) and trio copy number variant sequencing (trio-CNVseq) were used to effectively screen pathogenic variants of the patient. The detailed method were described in our previous work (Gao et al., 2019; Jiao et al., 2019). For trio-WES, total genomic DNA was extracted from the peripheral blood of the patient and her parents. DNA was randomly broken by ultrasonic treatment, followed by hybridization, enrichment, and sequencing on the Illumina HiSeq 2000 platform. The raw data were processed by fastp v0.18.1, and the sequences were aligned to the Ensemble GRCh37/hg19 reference genome. Single nucleotide polymorphism (SNP) and short indel calling and base quality score recalibration were conducted with GATK 3.8. High-quality and reliable mutation (>2X, mutation rate >10%, and mutation quality > 20) filtering was carried out with SAMtools 1.6. Annovar was used for variant annotation, and Provean, Polypen2_hdiv, Polypen2_hvar, MutationSotter, Revel, Sift, and M-Cap were performed for protein biological function prediction. Minor allele frequency (MAF) was against the 1000 Genomes Project database, hapmap, ExAC database, dbSNP, and NHLBI. A 0.1% cutoff of MAF was used for risk gene identification. The variants were classified into five categories using the method described by the American College of Medical Genetics and Genomics and the Association for Molecular Pathology (Richards et al., 2015).

The sequencing operation steps of trio-CNVseq are consistent with those of trio-WES. The clean data were then blasted to the human reference genome (hg19). PCR duplications were removed by Picard MarkDuplicates (Ebbert et al., 2016). The mixture-hidden Markov model (m-HMM) approach was used to estimate window-based copy number change points and copy number states. An in-house pipeline was used for calling CNVs larger than 100 kb.

Candidate CNVs were annotated by the genes within the CNVs and CNV intervals with constitutional CNV interpretation (Kearney et al., 2011).

### Sanger Sequencing

A DNA fragment covering the variant site was amplified using peripheral blood DNA. The primers 5’−CAATAACAACAACAGCCACCATCA−3’ (forward) and 5’−ATCATCGACGAGTACATCAAGGAG−3’ (reverse) were used to generate a product 448 bp in length. Amplification was performed with an annealing temperature of 60°C. The PCR products were then sequenced by ABI 3730XL (Thermo Fisher Scientific Inc., Waltham, USA) and analyzed using DNASTAR 5.0 software (DNASTAR, Inc., Madison, USA). The results were visualized with SnapGene Viewer (https://www.snapgene.com/snapgene-viewer/).

### Structure Modeling

Sequence alignment was performed using the online server UniProt (https://www.uniprot.org/), and the results were displayed with the online server ESPript3 (http://espript.ibcp.fr/ESPript/ESPript/). The crystal structures of Mus musculus RelB at different complex statuses (2V2T, 3DO7) were downloaded from PDB (http://www.rcsb.org/). Structure visualization was conducted using PyMOL software (http://www.pymol.org/).

### Reverse Transcription-Polymerase Chain Reaction

Total RNA was extracted from peripheral blood cells of the patient, her father, and two of the patient’s peers using Trizol bought from TIANGEN Biotech (Beijing) Co., Ltd. Amplification was performed with the primers of 5’−GAGAGCAGCACCGAGGCCAGC−3’ (forward) and 5’−CGTCGGTGCAGTCTTTCCCCA−3’ (reverse). The reaction was performed at 94°C for 10 min, followed by 40 cycles at 95°C for 5 s, 60°C for 15 s, and 72°C for 10 s, and a final extension at 72°C for 10 min. Bands intensities were determined by comparison to those of GAPDH.

### Western Blotting

Whole blood leukocytes were extracted from the blood of the patient, her father, and two of the patient’s peers for healthy controls. Then, the cells were homogenized with RIPA buffer (Beyotime Biotechnology, China). Protein concentration was determined with the BCA protein assay kit strictly according to the protocol provided by the manufacturer (Beyotime Biotechnology, China). A total of 50 μg of protein was loaded into 10% SDS-PAGE gels followed by transfer onto Merck Millipore PVDF membranes (Billerica, MA, USA). After blocking with 5% skimmed milk, the membranes were incubated with primary antibodies against RelB or β-actin (1:2000, Abcam, Cambridge, MA, USA) at 4°C overnight followed by another 1-h incubation with secondary antibody (1:2000, Abcam, Cambridge, MA, USA). The epitope recognized by the anti-RELB antibody is the C terminal Human RelB amino acids 550–650. Protein bands were visualized by the BeyoECL plus kit (Beyotime Biotechnology, China), and ImageJ was used to analyze the images.

### Flow Cytometry

Peripheral blood mononuclear cells (PBMCs) were isolated from peripheral blood sample collected from the patient. The cells were then used for flow cytometric analysis according to the manufacturer’s instructions (Mindray, Shenzhen, China), the relevant kits were used for flow cytometry to measure the surface phenotype of immune cells. Anti-CD3 was purchased from Beckman Coulter (Miami, FL, USA) or BD Biosciences (San Jose, CA, USA). Antibodies of CD45, IFN-γ, IL-4, CD28, CD38, CD45RA, CD45, CD4, CD25, and FoxP3 Alexa Fluor^®^ 647 were provided by BD Pharmingen (San Diego, CA, USA). Antibodies of CD4, CD45, CD8, CD4, and CD3 were bought from BD Biosciences (San Jose, CA, USA). Anti-CD8, anti-CCR7, and anti-HLA-DR were from BioLegend (San Diego, CA, USA). Anti-IL-17 was provided by eBioscience (San Diego, CA, USA).

## Results

### Case Report

An 8-month-old female infant developed intermittent high fever without obvious inducement; the highest body temperature was 40.7°C. She had paroxysmal cough with sputum in the throat, without asthma, shortness of breath and cyanosis. The fever was repeated every 6–7 h. Two days later, she went to a local hospital and received symptomatic treatment. After treatment for 2 days, she developed shortness of breath and irritability, so she received high-flow oxygen inhalation, anti-infection treatment with meropenem and linezolid, and immune support with human immunoglobulin (7.5 g). However, instead of improving, her situation continued to deteriorate. The baby was in critical condition and was transferred to our hospital.

**Figure 1:**
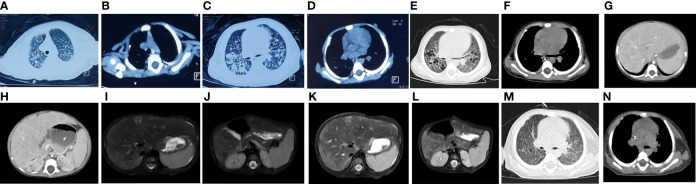
Imaging results for the patient. CT showed that the transmittance of both lungs decreased, and there were many high-density nodular lesions with clear boundaries and cavities in both lungs **(A–D)** and enhanced CT scan of the chest and abdomen showed thymic dysplasia, diffuse pulmonary nodules **(E, F)** and liver nodules **(G, H)**. Abdominal MRI showed many short T2 signal sites in **(I, J)** liver and kidney **(K, L)**. The original bilateral lung fields were absorbed after treatment **(M, N)**.

The patient’s growth and development were consistent with her peers. She was in poor spirits and had no rash or abnormal secretion from the external auditory canal. Her pulse oxygen saturation (SpO2) was maintained at 95% or above during oxygen inhalation. Breathing was shallow and fast, with a breathing frequency of 50 times/min, and the three concave signs were positive. No obvious rales were heard. CT showed that the transmittance of both lungs decreased, and there were many high-density nodular lesions with clear boundaries and cavities in both lungs (**Figures 1A–D**). Her thymus was small (**Supplementary Figure 1**), which may have led to an abnormal number and function of T lymphocytes, suggesting the possibility of immunodeficiency. Laboratory tests in local hospitals showed that hemoglobin (HGB) decreased significantly (75 g/L, normal range: 113–151 g/L). The white blood cell count was not high (8.74*10^9/L, normal range: 3.69–9.16*10^9/L); however, CRP (124.8 mg/L, normal range: 0–6 mg/L) and PCT (5.26 nil/ml, normal range: 0-0.5 nil/ml) increased significantly. The liver and spleen were palpable, 6 and 5 cm under the rib, respectively. Consistently, liver function tests showed that alanine aminotransferase (ALT, 158 U/L, normal range: 7–40 U/L), aspartate aminotransferase (AST, 560 U/L, normal range: 13–35 U/L), glutamyl transferase (GGT, 661 U/L, normal range: 7–45 U/L), and alkaline phosphatase (ALP, 316 U/L, normal range: 35–100 U/L) were increased, which indicated that the liver was damaged.

Combined with the symptoms and related examination results, the infant was diagnosed with severe pneumonia, respiratory insufficiency, sepsis, moderate anemia, and liver function damage. The anti-infective drugs administered were as follows: amphotericin B, voriconazole, vancomycin, and cefoperazone sulbactam. During the treatment period, the patient’s body temperature increased, and a rash appeared (**Supplementary Figure 2**). After stopped vancomycin and intravenous injection of methylprednisolone (2 mg/kg), the rash disappeared indicating drug-induced hypersensitivity syndrome, which might be due to immunological overreaction. Finally, the baby recovered and was discharged from the hospital after being treated for 2 and a half months. Two weeks later, the reexamination showed that there was no *T. marneffei* infection recurrence. However, follow-up showed that she suffered from repeated infections, such as Salmonella infection.

### Laboratory Examination Results Indicated Infection

To dynamically monitor the progress of the disease and provide a reference index for drug selection and therapeutic effects, we conducted routine blood tests. The detailed test results are shown in **Supplementary Table 1**. Her hemoglobin decreased during admission. Her white blood cell count, neutrophil count, and neutrophil ratio were normal or elevated. The numbers of circulating lymphocytes were normal, but the percentage of circulating lymphocytes was sometimes decreased. Tests on June 2 and June 28 showed increases in both PCT and CRP, indicating infection. Liver and kidney function examinations showed that the related injury indicators gradually decreased (**Supplementary Table 1**), indicating that the liver injury gradually recovered. Blood gas analysis showed that her respiratory function and acid-based balance were generally normal during oxygen inhalation.

### Imaging Examination Demonstrated the Potential Existence of Fungal Infection and Immunodeficiency

In order to understand and dynamically grasp the changes in the chest and abdominal organs of the patient, we carried out relevant imaging examinations. Ultrasonic examination of the heart and abdomen showed patent foramen ovale, enlarged liver, enlarged spleen and ascites. An enhanced CT scan of the chest and abdomen showed thymic dysplasia, diffuse pulmonary nodules and liver nodules, and many balloon-like air sacs in both lungs, which was consistent with special features of fungal infection (**Figures 1E–H**). Abdominal MRI showed that the intensities of the liver and kidney parenchyma were uneven, and there were many short T2 signal sites of unknown nature, suggesting that an infectious disease was present (**Figures 1I–L**). After a few days of treatment chest CT showed that the patchy high-density lesions scattered in the original bilateral lung fields were absorbed, the diaphragm surface was smooth, and the diffuse nodules in both lungs had decreased (**Figures 1M, N**). The balloon-like air sacs in both lungs were significantly improved. Together, the imaging examinations highly suggested the existence of fungal or tubercle bacillus infection.

### Biological Inspection of Pathogens Demonstrated *T. marneffei* Infection

**Figure 2:**
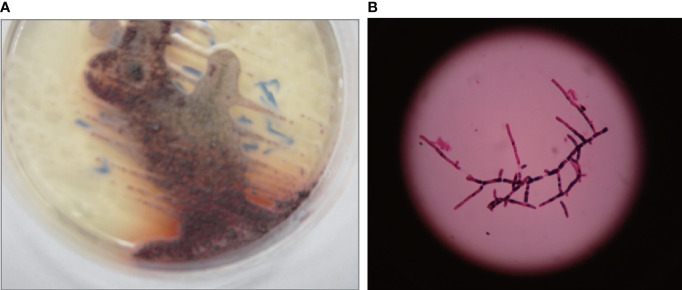
Representative images of *Talaromyces marneffei* colonies. Clones appeared on the 3^rd^ day when cultured in Sabouraud medium at 28°C **(A)**. A light grey-brown film with a diameter of approximately 3–5 mm was noted. A typical red product developed. A clone cultured at 28°C was selected to prepare a bacterial smear and was observed under a high-magnification microscope **(B)**. Branched, colorless and transparent mycelium was observed.

The child had respiratory tract infection symptoms and abnormal lung imaging. To understand the etiology of the disease, a series of related pathogens were tested. HIV, toxoplasma, rubella, cytomegalovirus, herpes simplex viruses, influenza A, influenza B, respiratory syncytial virus, adenovirus, parainfluenza types 1, 2, and 3, 2019-nCoV, Mycoplasma pneumoniae, and tubercle bacillus tests were all negative. No pathogenic bacteria or fungi were found in sputum culture. The values of (1,3)-β-D-glucan (G experimental) and galactomannan (GM) detection by ELISA were 295.52 pg/ml (normal: 0–70 pg/ml) and 0.45 μg/L (normal: 0–0.65 μg/L), respectively. Bone marrow and blood cultures showed *T. marneffei* infection (**Figure 2**), and reexaminations after treatment were normal. No pathogenic bacteria or fungi were found in the patient’s blood culture on after treated for 1 month. In summary, biological detection of pathogens showed that the patient was HIV-negative but infected with *T. marneffei*.

### Immune System Evaluation Showed Immunodeficiency

**Figure 3:**
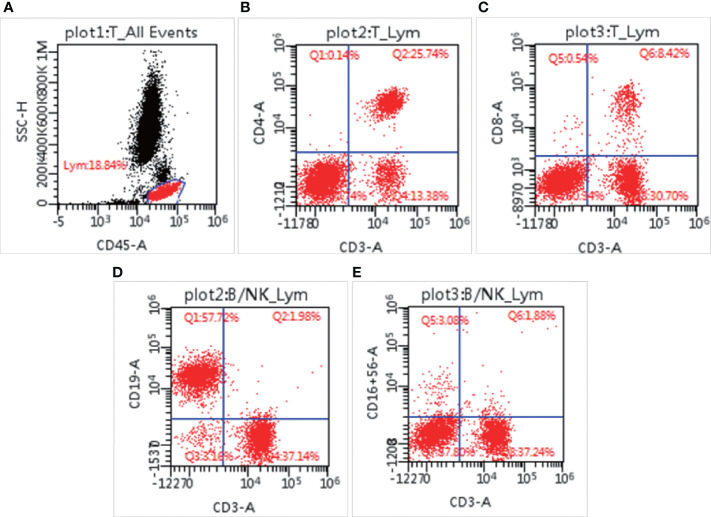
Lymphocyte subsets measured by flow cytometry. The percentage of lymphocytes in blood cells **(A)**, CD3^+^CD4^+^ positive cells **(B)**, CD3^+^CD8^+^ positive cells **(C)**, CD3^-^CD19^+^ cells **(D)**, and CD3^-^CD16^+^CD56^+^ cells **(E)**.

*T. marneffei* infection is usually accompanied by immunodeficiency. To understand the damage to her immune system, we conducted relevant immunological experiments. ELISA showed that her IgE was 25.75 IU/ml, which ruled out the possibility of hyperimmunoglobulin E syndrome. Turbidimetry assay on Jun 3 showed that IgA (0.081 g/L, normal range: 0.7–4 g/L) had decreased, IgG (20.5 g/L, normal range: 7–16 g/L) had increased, and IgM was in the normal range (0.76 g/L, normal range: 0.4–2.3 g/L). The increase in IgG may have been caused by intravenous immunoglobulin. Turbidimetry assays indicated that complement C3 and complement C4 were 0.62 g/L (normal range: 0.9–1.8 g/L) and 0.35 g/L (normal range: 0.1–0.4 g/L), respectively, indicating that the complement system was normal. To understand the changes in different immune subsets in detail, flow cytometry was used for analysis. As shown in **Supplementary Table 1**, total lymphocyte count (521/μl, normal range: 723–2737/μl), CD3+ T lymphocyte count (521/μl, normal range: 723–2737/μl), CD3+CD4+ T lymphocyte count (338/μl, normal range: 404–1612/μl), CD3+CD8+ T lymphocyte count (111/μl, normal range: 220–1129/μl), and NK cell count (42/μl, normal range: 84–724/μl) had decreased, while the number of CD19 positive B cells (778/μl, normal range: 80–316/μl) had increased (**Figure 3**). It is worth noting that although the patient’s CD3+CD4+ T cells (338/μl, normal range: 404–1612/μl) had decreased, the ratio of CD3+CD4:CD3+CD8+T cells (3.06, normal range: 1.8–2.5/1) had increased. The patient’s respiratory burst activity was normal, and the neutrophil activation rate was 99.97%. The neutrophil activation rates of the father and mother were 99.99% and 100.0%, respectively. In summary, our results demonstrated that the patient had an impaired immune system.

### Trio-WES Identified a Homozygous Variant of *RELB* as the Candidate Etiology

The patient was HIV-negative, infected with *T. marneffei*, and had an impaired immune system. To determine the cause of immune injury, trio-WES and trio-CNVseq were performed. Trio-CNVseq did not find any possible pathogenic variant. Analysis using trio-WES data (**Supplementary Table 2**) was then performed as described previously (Zhang X. et al., 2020). We first evaluated an autosomal recessive disorder and searched for compound heterozygous or homozygous variants on the autosomes. Finally, homozygous variation (variation depth/total depth: 17/17) of NM_006509: c.400_c.401insAGC/p.Lys134 delinsLysGln in *RELB* was identified in the patient. Her father (variation depth/total depth: 7/19) and mother (variation depth/total depth: 9/19) were heterozygote for this site. This variant has not been recorded in any published single nucleotide polymorphism (SNP) database (dbSNP, 1000 Genomes, HGMD, ESP, ExAC, Clinvar) or OMIM. The prediction of protein function indicates that the variant will lead to a change in protein length. Combined Annotation Dependent Depletion (CADD, https://cadd.gs.washington.edu/) showed that the PHRED for the variant of the patient was 18.80. The interpretation of the ACMG guidelines is PM2+PM4, which met the standard of “uncertain significance.” The clinical features of our patient were similar to those reported by Roifman et al. (Sharfe et al., 2015); therefore, *RELB* was considered to be the candidate pathogenic gene.

### Sanger Sequencing Validated the Variant

**Figure 4:**
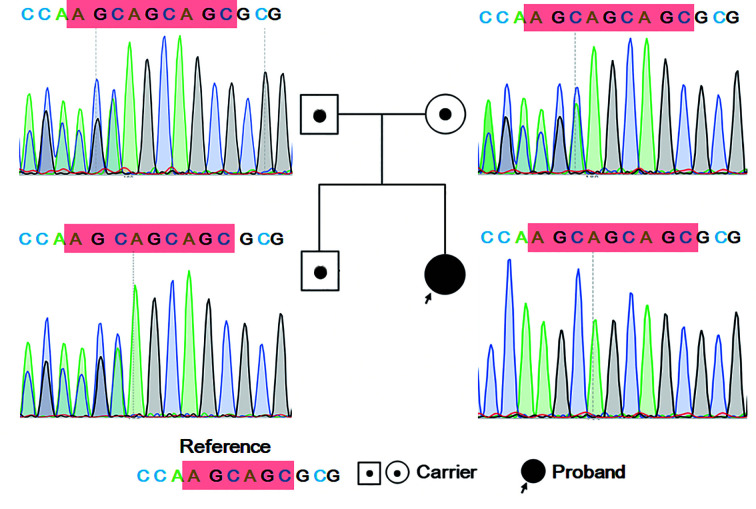
Sanger sequencing result for the family. The proband has homozygous variation of NM_006509: c.400_c.401insAGC/p. Lys134 delinsLysGln in *RELB*. Her parents and elder brother have heterozygous mutations in this region.

To validate the results of trio-WES, Sanger sequencing was performed. As shown in **Figure 4**, the proband had a homozygous insertion variant of AGC in the *RELB* gene, while her parents and elder brother had a heterozygous mutation at this locus. The results confirmed that the candidate pathogenic variants were passed from the parents.

### *In Silico* Structural Modeling Indicated That the Variant May Be Pathogenic

**Figure 5:**
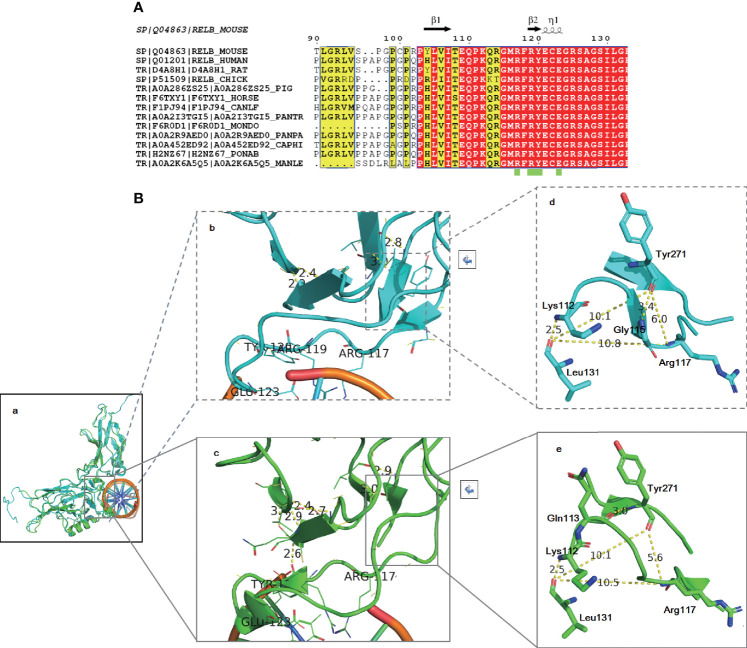
Amino acid alignment **(A)** and structural modeling **(B)** demonstrated that the identified variant may be pathogenic. Amino acid alignment showed that the region of RelB around the identified variant is highly conserved among species **(A)**. The RelB structure is highly conservative (Ba). The local secondary structures around the DNA binding region are slightly different: R114-M116 form a β sheet in 3do7 (Bb) and C122-G124 make up an α helix in 2v2t (Bc). However, the trend of amino acids is completely consistent. The distances between K117, L131, and Y271 are almost the same in two different states (Bd, Be).

Protein biological function prediction showed that the variant could lead to a change in protein length, but this strategy could not directly predict protein function damage. To analyze the possibility of this mutation as a pathogenic factor, structural modeling was performed. As shown in **Figure 5A**, sequence alignment showed that the identified region of RelB is close to the DNA-binding domain (green box), which is highly conserved in the evolution between species, indicating low tolerance to mutation. As there is no human RelB structure covering the identified region, Mus musculus RelB structures (2o61 and 3do7) were used. The root mean square deviation (RMSD) of 3do7 (cyan) to 2v2t (green) is 0.927, indicating that the structure of RelB is highly conserved (**Figure 5Ba**). R114-M116 form a β sheet in 3do7 (**Figure 5Bb**) but not 2v2t (**Figure 5Bc**). C122-G124 make up an α helix in 2v2t (**Figure 5Bc**), but there is no such helix in 3do7 (**Figure 5Bb**). It is worth noting that although the local secondary structures are slightly different, the trends of amino acids are completely consistent (**Figures 5Bb, Bc**). The amino acid residues K112 and L131 form a 2.5 Å hydrogen bond in both structures (**Figures 5Bd, Be**). The amino acid residue Y271 forms a hydrogen bond with G116 in 3do7 and with Q113 in 2v2t. The amino acid residue K117 is involved in binding with DNA (**Figure 5A**). As shown in **Figures 5Bd** and **Be**, the amino acid distances between K117, L131, and Y271 are essentially the same in two different states. Therefore, the space in this area is relatively stable. This limited region can only accommodate four amino acids (Q113-M116). Inserting another Gln after K112 will lead to the destruction of this stable local structure, thus affecting protein function. In conclusion, structural modeling highly suggested that the identified mutation in RelB might be pathogenic.

### RelB Expression Decreased in the Patient

**Figure 6:**
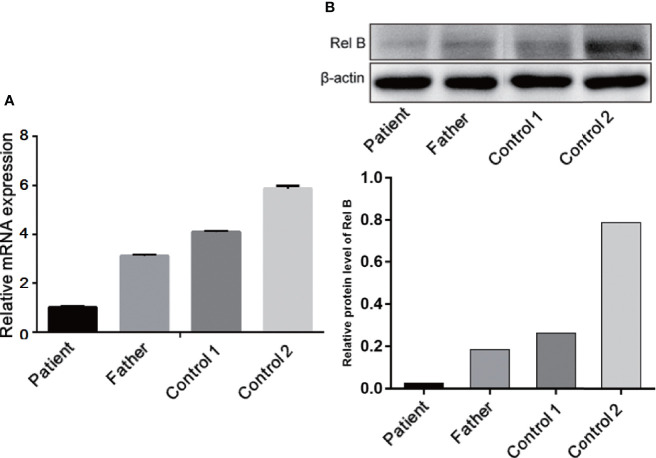
Expression of RelB at the mRNA **(A)** and protein levels **(B)** showed that the RelB expression level in the patient was significantly lower than that in her father and the healthy controls of her peers.

To investigate the expression of RelB, RT-PCR and Western blotting was performed using whole blood leukocytes extracted from the patient, her father, and two of the patient’s peers as healthy controls. As shown in **Figure 6**, *RELB* expression in the patient was lower than her father and health controls. Consistent with mRNA result, the RelB expression level in the patient (RelB/β-actin, 0.026) was seven times lower than that in her father (RelB/β-actin, 0.185) and was at least ten times lower than that in the healthy controls (RelB/β-actin, 0.26 and 0.79).

### Re-Examination of Immunologic Function

**Figure 7:**
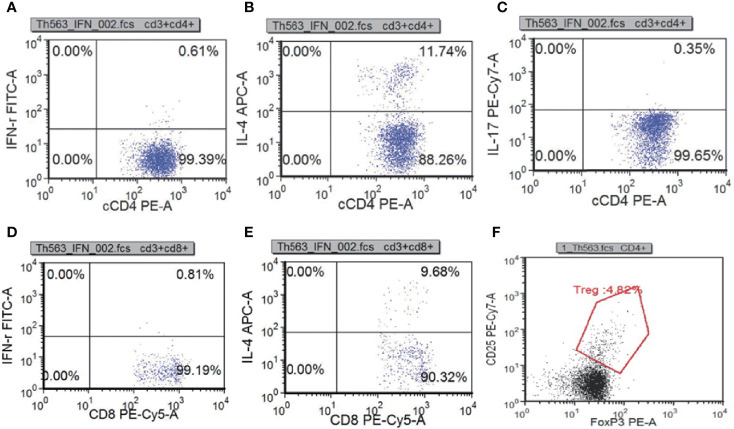
T subset assays reexamination results measured by flow cytometry. Th1(IFN-γ+CD4+):CD4 **(A)**, Th2(IL-4+CD4+):CD4 **(B)**, Th17(IL-17+CD4+):CD4 **(C)**, Tc1(IFN-γ+CD8+): CD8 **(D)**, Tc2(IL-4+CD8+):CD8 **(E)** Treg(CD25+FOXP3+):CD4+ **(F)**.

**Figure 8:**
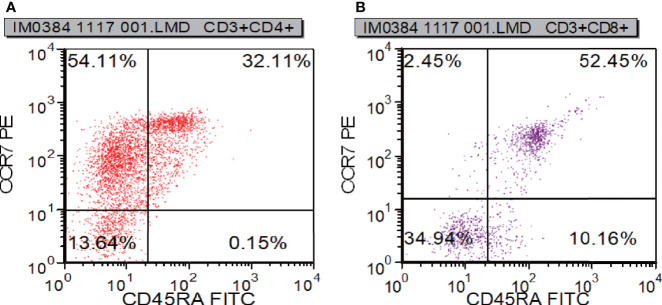
T cell functional analysis results measured by flow cytometry. CR7+CD45RA+ cells **(A)**, CCR7-CD45RA+ cells **(A)**, and CCR7-CD45RA- cells **(B)** decreased whereas CCR7+CD45RA- cells **(B)**.

To learn about immunocyte function, T subset assays including Th1, Th2, Th17, TN, TEM, TERMA, and TCM assays for CD3+CD4+ and CD3+CD8+ were performed during re-examination. As shown in **Figure 7** and **Supplementary Table 3**, Th1, Th17, Treg, TH1/TH2, and TH17/Treg were decreased, indicating that the immune function of T cells was impaired. CR7+CD45RA+ cells (32.11% of CD4; normal range, 60%–80%), CCR7-CD45RA+ cells (0.15% of CD4; normal range, 0.18%–15%), and CCR7-CD45RA- cells (13.64% of CD4; normal range: 15%–25%) decreased whereas CCR7+CD45RA- cells (54.11% of CD4; normal range, 10%–25%) increased (**Figure 8**). This indicated that most of the memory T cells in children are central memory cells, but not effector memory cells. Routine immune detection using immunoturbidimetry showed that the main functions of B lymphocytes were essentially normal compared with those of her peers.

### Discussion

Here, we report a Chinese infant with CID due to the NM_006509: c.400_c.401insAGC/p. Lys134delinsLysGln homozygous mutation in the *RELB* gene. She showed recurrent infections. To the best of our knowledge, this is the fourth case confirming *RELB* as one of the genes causing CID with bi-allelic disruption. Biological tests on pathogens showed that she was HIV-negative but was infected with *T. marneffei*. This is the first report on *T. marneffei* pulmonary infection in a patient with a *RELB* mutation.

The main manifestations of *T. marneffei* infection are fever, weight loss, lymphadenectasis, anemia, and hepatosplenomegaly, but they are nonspecific and have no significance for differential diagnosis (Vanittanakom and Sirisanthana, 1997; Li et al., 2016; Zhang W. et al., 2020). Our patient developed fever, anemia and hepatosplenomegaly. She showed no signs of weight loss, which might be mainly due to the short course of disease. The baby suffered from severe pneumonia, with repeated fever, but there were no obvious rales. She had hepatosplenomegaly, and lung CT and abdominal CT/MRI showed multiple nodule changes. CRP and PCT were significantly increased, clinically suggesting the possibility of a blood-borne infection. Staphylococcus aureus and fungal infections are common. Therefore, vancomycin and amphotericin B were selected as anti-infective drugs when the patient was admitted to the hospital, and her blood and bone marrow cultures improved immediately. Soon, the culture results showed *T. marneffei* infection.

Trio-WES identified the NM_006509: c.400_c.401insAGC/p. Lys134delinsLysGln homozygous variant in *RELB*. Sanger sequencing revealed that the variant was inherited from the parents. Using a candidate gene approach, Lee *et al.* performed direct sequencing by PCR in four HIV-negative patients infected with *T. marneffei*; however, the results were negative for most of the patients (Lee et al., 2012). We previously showed that the combination of WES and CNVseq enables the diagnosis of rare neurological disorders (Jiao et al., 2019). The current report indicates that NGS should also be recommended for those with undiagnosed immune abnormalities (Sun et al., 2020; Zhang X. et al., 2020).

We and other research groups have analyzed gene mutation points *in silico* by using the solved protein structure (Heinen et al., 2016; Li et al., 2019). Sequence alignment showed that the mutation site is close to the DNA-binding domain, which is highly conserved among species. The insertion of extra amino acids will destroy this local structure, leading to the loss of protein function. The reported patients had a premature stop codon mutation, and mRNA expression in them was lower than that of health controls (Mericoa et al., 2015). In light of this, the authors consider that the variant might cause a “knockout” phenotype (Mericoa et al., 2015). Consistent with the reported result, our RT-PCR and Western blot analysis showed that the expression of RelB in the patient was lower than that in her father and peer controls at both mRNA and protein levels. Compared with the peer controls, the ratio of the protein of the patient was much lower than mRNA, indicating that the mutated protein may be unstable. We consider that our variant might lead to protein degradation, hence causing a “knockout” phenotype. Further studies are needed to reveal the pathogenesis the mutation.

The reported patients had symptoms of pneumonia, otitis media, vesicular rash and urinary tract infections, while our patient had symptoms of pneumonia (Sharfe et al., 2015). Therefore, repeated respiratory tract infection is the main clinical feature of RelB deficiency. Compared with the controls, circulating lymphocytes in the patients showed normal to increased numbers, with an elevated CD4^+^:CD8^+^ ratio (Sharfe et al., 2015). Consistent with the previous report, the surface phenotype of our patients determined by flow cytometry analysis showed an increase in the CD4^+^:CD8^+^ ratio. In the reported patients, the number of CD4^+^ T cells was increased or normal, but in our patient, CD4^+^ T cells were decreased (Sharfe et al., 2015). It is not difficult to understand this phenomenon because the reported trend of T cells in patients is not completely consistent, indicating that patients are heterogeneous. Re-examination of the T cell subset indicated that the patient had T cell functional deficiency. There are too few CD4+ lymphocytes available in the patient. When the infection occurs, the immediate memory response of T cells to drug therapy may be inhibited, which is consistent with that of Roifman et al. (Sharfe et al., 2015).Our patient did not have a thymus biopsy. However, a CT scan showed decreased thymus. Combining the results of flow cytometry and CT, we can conclude that this patient has T cell immunodeficiency. The number of CD3^-^CD19^+^ cells greatly increased; however, the concentration of immunoglobulin was normal during treatment, suggesting that B cells had functional defects. In view of these results, we concluded that the patient suffered from CID. According to clinical symptoms, imaging, immunology, pathogen biology and gene sequencing results, *RELB* mutation was considered to be the etiology for the patient. Immunoglobulin replacement and early empirical antibiotic treatment are generally used for patients with CID. Hematopoietic stem cell transplantation (HSCT) was performed for two patients by Roifman et al., and the outcome was good (Ovadia et al., 2017). In light of this, we are still considering HSCT for the patient because she has symptoms of repeated infection.

Human genetic diseases affecting a large number of NF-κB components have been reviewed by Zhang et al. (2017). Deficiencies that disrupt NF-κB cause TNF-induced apoptosis to predominate, which impairs immunity. RelB is involved in the alternative pathway of NF-κB; therefore, the patient showed CID. In addition to *RELB*, *STAT3, STAT1, TNFSF5 (CD40L)*, and *IFNGR1* mutations (**Supplementary Table 4**) are found in HIV-negative patients infected with *T. marneffei* (Lee et al., 2012; Lee et al., 2014; Fan et al., 2018; Lee et al., 2019; Zhang W. et al., 2020). Although some patients have *T. marneffei* infection only after adulthood, these patients with immunodeficiency caused by gene mutations all have clinica\l symptoms in the early stage. This indicates that we should consider the possibility of fungal infection in patients with infections that are difficult to diagnose who showed evidence of immunocompromise shortly after birth.

## Data Availability Statement

The data presented in the study are deposited in the NCBI ClinVar (https://www.ncbi.nlm.nih.gov/clinvar/) repository, accession number SCV001473636.

## Ethics Statement

The studies involving human participants were reviewed and approved by Medical Ethics Committee in Hunan Provincial People’s Hospital. Written informed consent to participate in this study was provided by the participants’ legal guardian/next of kin. Written informed consent was obtained from the minor(s)’ legal guardian/next of kin for the publication of any potentially identifiable images or data included in this article.

## Author Contribution

X-AY: conceived and designed the study project, performed data analysis, and wrote the manuscript. XD and HH: conceived and designed the study project, performed clinical diagnosis, data analysis, and collected the samples. LZ, MC, FP, BZ, and XC: participated in data entry. All authors contributed to the article and approved the submitted version.

## Funding

This study was supported by operating fund of Hunan Provincial Key Laboratory of Pediatric Respirology (2019TP1403), Technology Innovation Guidance Project-Science and Technology Work Conference, Hebei Key Laboratory of Nerve Injury and Repair, Natural Science Foundation of Province (No. H2020406049), and Scientific and Technological Research Projects of Hebei Higher Education (No. ZD2019084).

## Conflict of Interest

The authors declare that the research was conducted in the absence of any commercial or financial relationships that could be construed as a potential conflict of interest.

## Supplementary Material

Supplementary Figure 1 The specific value of thymus (arrow). The right width and thickness are 8.46 and 15.71 mm; the left width and thickness are 12.35 and 18.38 mm. According to the standard thymus size of normal children of the same age in China, the left thymus (normal: width, 12.69 ± 3.25; thickness, 28.57 ± 5.75) is slightly decreased, while the right thymus (normal: width, 12.4 ± 3.25; thickness, 23.34 ± 5.41) is obviously reduced.

Supplementary Figure 2 During the treatment period, a red miliary rash appeared on the patient’s trunk, which was higher than the skin surface and faded with pressing.
