# Supplementary figures and images for: Disseminated Talaromyces marneffei Infection in a Non-HIV Infant With a Homozygous Private Variant of RELB
Source: Front Cell Infect Microbiol. 2021 Mar 15;11:605589. doi: 10.3389/fcimb.2021.605589 (PMC8005656; doi:10.3389/fcimb.2021.605589)

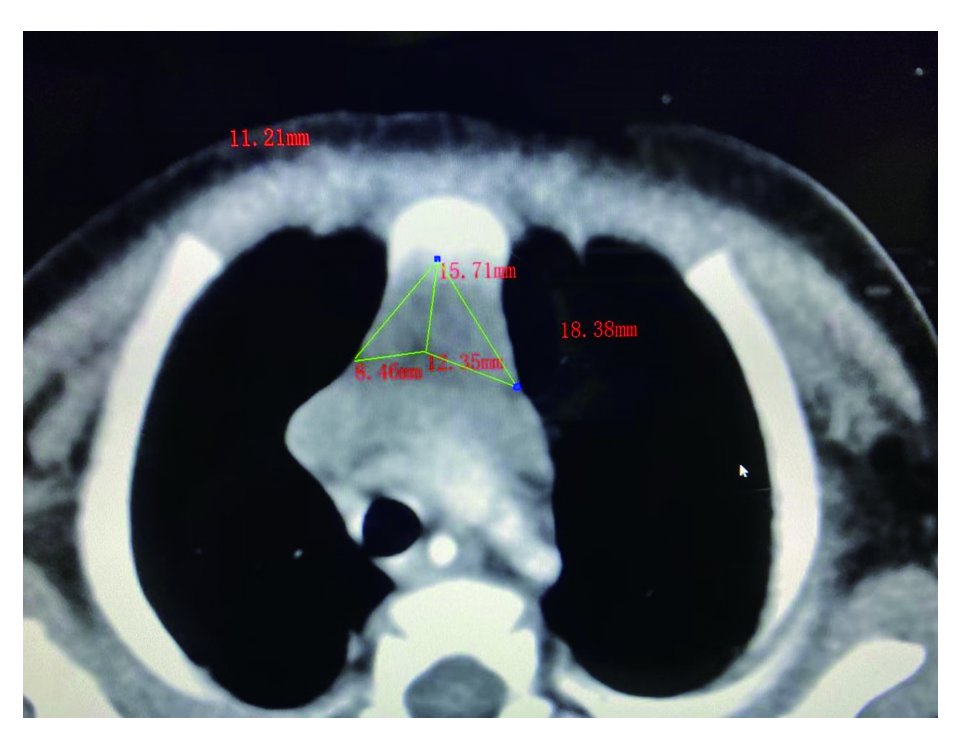

Supplement: Supplementary Figure 1 — The specific value of thymus (arrow). The right width and thickness are 8.46 and 15.71 mm; the left width and thickness are 12.35 and 18.38 mm. According to the standard thymus size of normal children of the same age in China, the left thymus (normal: width, 12.69 ± 3.25; thickness, 28.57 ± 5.75) is slightly decreased, while the right thymus (normal: width, 12.4 ± 3.25; thickness, 23.34 ± 5.41) is obviously reduced. [file Image_1.tif]

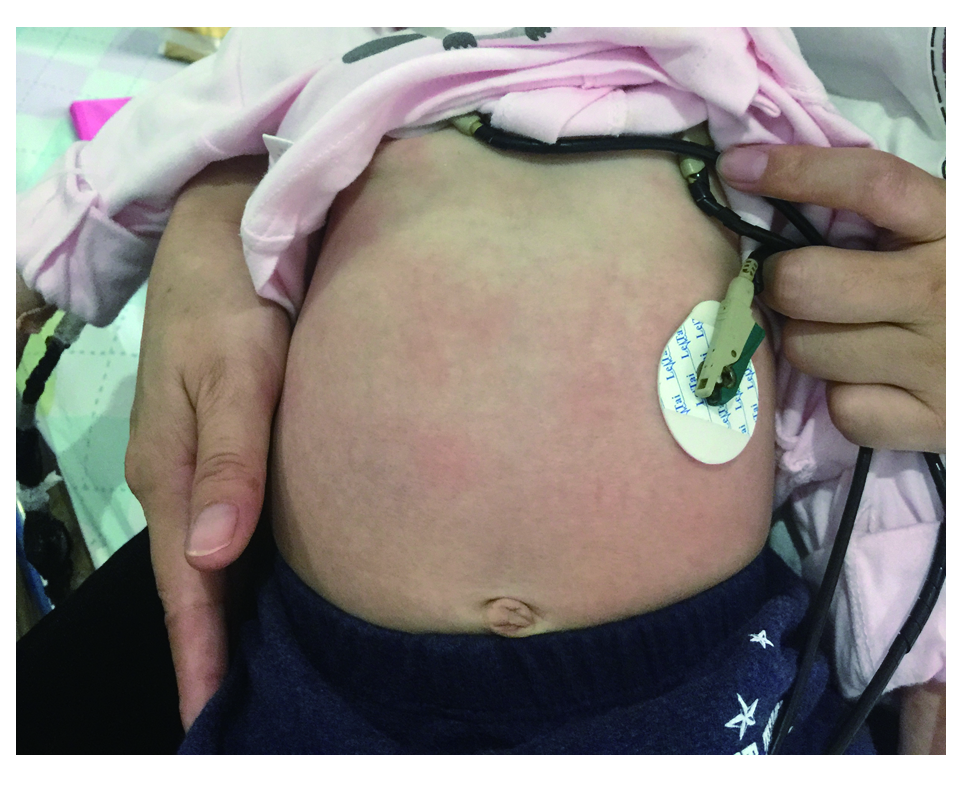

Supplement: Supplementary Figure 2 — During the treatment period, a red miliary rash appeared on the patient’s trunk, which was higher than the skin surface and faded with pressing. [file Image_2.tif]
