# Supplementary material for: Disseminated Talaromyces marneffei Infection in a Non-HIV Infant With a Homozygous Private Variant of RELB
Source: Front Cell Infect Microbiol. 2021 Mar 15;11:605589. doi: 10.3389/fcimb.2021.605589 (PMC8005656; doi:10.3389/fcimb.2021.605589)
Supplement: Supplementary file 5 [file Table_3.docx]

**Supplementary Table 3** Results of T cell functional subsets by flow cytometry and immunoturbidimetry method

| Index | Vale | Indication | Reference |
| --- | --- | --- | --- |
| Th1(IFN-γ+CD4+):CD4+ | 0.61 % | ↓ | 0.95-26.00 |
| Th2(IL-4+CD4+):CD4+ | 11.74 % | ↑ | 0.61-3.00 |
| Th17(IL-17+CD4+):CD4+ | 0.35 % | ↓ | 0.44-2.01 |
| Tc1(IFN-γ+CD8+):CD8+ | 0.81 % |  |  |
| Tc2(IL-4+CD8+):CD8+ | 9.68 % |  |  |
| Treg(CD25+FOXP3+):CD4+ | 4.82 % | ↓ | 5.10-12.70 |
| Th1(IFN-γ+CD4+) | 9.00 /μl |  | 5.52-182.00 |
| Th2(IL-4+CD4+) | 174.00 /μl | ↑ | 4.04-21.00 |
| Th17(IL-17+CD4+) | 5.00 /μl |  | 3.07-14.00 |
| Tc1(IFN-γ+CD8+) | 2.00 /μl |  |  |
| Tc2(IL-4+CD8+) | 25.00 /μl |  |  |
| Treg(CD25+FOXP3+) | 71.00 /μl |  | 28.00-142.00 |
| Th1:Th2 | 0.05 | ↓ | 0.73-18.50 |
| Th17:Treg | 0.07 | ↓ | 0.09-0.47 |
| CD3+:LYM | 47.05 % | ↓ | 53.70-82.80 |
| CD3+CD4+:CD3+ | 68.45 % |  | 42.60-78.00 |
| CD3+CD8+:CD3+ | 20.97 % |  | 14.80-48.40 |
| CD3+CD4+CCR7+CD45RA+:CD4+ | 32.11 % |  | 7.20-68.90 |
| CD3+CD4+CCR7+CD45RA-:CD4+ | 54.11 % |  | 15.00-64.30 |
| CD3+CD4+CCR7-CD45RA+:CD4+ | 0.15 % | ↓ | 0.16-14.41 |
| CD3+CD4+CCR7-CD45RA-:CD4+ | 13.64 % |  | 3.13-24.83 |
| CD3+CD8+CCR7+CD45RA+:CD8+ | 52.45 % |  | 2.60-72.40 |
| CD3+CD8+CCR7+CD45RA-:CD8+ | 2.45 % | ↓ | 2.70-36.20 |
| CD3+CD8+CCR7-CD45RA+:CD8+ | 10.16 % |  | 1.60-62.00 |
| CD3+CD8+CCR7-CD45RA-:CD8+ | 34.94 % |  | 4.70-60.10 |
| CD3+HLADR+:CD3+ | 7.54 % |  | 1.40-21.60 |
| CD3+CD28+:CD3+ | 91.40 % |  |  |
| CD3+CD38+:CD3+ | 6.61 % |  | 5.98-26.80 |
| CD3+CD4+HLADR+:CD4+ | 6.42 % |  | 5.42-32.78 |
| CD3+CD4+CD28+:CD4+ | 99.78 % |  | 73.77-99.97 |
| CD3+CD4+CD38+:CD4+ | 4.31 % | ↓ | 6.13-32.20 |
| CD3+CD8+HLADR+:CD8+ | 9.88 % | ↓ | 9.97-71.53 |
| CD3+CD8+CD28+:CD8+ | 80.64 % |  | 26.41-88.91 |
| CD3+CD8+CD38+:CD8+ | 11.49 % | ↑ | 0.93-7.03 |
| CD3+:LYM | 1646.75 /μl |  | 270.00-2586.00 |
| CD3+CD4+:CD3+ | 1127.20 /μl |  | 199.00-1414.00 |
| CD3+CD8+:CD3+ | 345.32 /μl |  | 61.00-1118.00 |
| CD3+CD4+CCR7+CD45RA+:CD4+ | 361.94 /μl |  | 4.00-1079.00 |
| CD3+CD4+CCR7+CD45RA-:CD4+ | 609.93 /μl |  | 70.00-671.00 |
| CD3+CD4+CCR7-CD45RA+:CD4+ | 1.69 /μl |  | 0.00-301.00 |
| CD3+CD4+CCR7-CD45RA-:CD4+  CD3+CD8+CCR7+CD45RA+:CD8+ | 153.75 /μl 181.12 /μl | ↑ | l0.00-141.00  18.00-355.00 |
| CD3+CD8+CCR7+CD45RA-:CD8+ | 8.46 /μl |  | 7.00-206.00 |
| CD3+CD8+CCR7-CD45RA+:CD8+ | 35.08 /μl |  | 8.00-635.00 |
| CD3+CD8+CCR7-CD45RA-:CD8+ | 120.66 /μl |  | 13.00-457.00 |
| CD3+HLADR+:CD3+ | 124.16 /μl | ↑ | 4.00-559.00 |
| CD3+CD28+:CD3+ | 1505.13 /μl |  |  |
| CD3+CD38+:CD3+ | 108.85 /μl |  | 102.00-554.00 |
| CD3+CD4+HLADR+:CD4+ | 72.37 /μl |  | 36.00-257.00 |
| CD3+CD4+CD28+:CD4+ | 1124.72 /μl |  | 333.00-1844.00 |
| CD3+CD4+CD38+:CD4+ | 48.58 /μl | ↓ | 69.00-547.00 |
| CD3+CD8+HLADR+:CD8+ | 34.12 /μl | ↓ | 41.00-547.00 |
| CD3+CD8+CD28+:CD8+ | 278.47 /μl |  | 82.00-949.00 |
| CD3+CD8+CD38+:CD8+ | 39.68 /μl |  | 13.00-124.00 |
| IgG | 9.77 g/L |  | 8-17 |
| IgA | 0.35 g/L | ↓ | 0.72-4.29 |
| IgM | 0.52 g/L | ↓ | 0.6-2.6 |
| C3 | 1.84 g/L |  | 0.78-2.1 |
| C4 | 0.72 g/L | ↑ | 0.17-0.48 |
