# Supplementary material for: Disseminated Talaromyces marneffei Infection in a Non-HIV Infant With a Homozygous Private Variant of RELB
Source: Front Cell Infect Microbiol. 2021 Mar 15;11:605589. doi: 10.3389/fcimb.2021.605589 (PMC8005656; doi:10.3389/fcimb.2021.605589)
Supplement: Supplementary file 6 [file Table_4.docx]

**Supplementary Table 4 Summary of mutations of genes found in HIV-negative patients infected with *T. marneffei* reported so far*.***

| Gene | Mutation site |
| --- | --- |
| STAT3 | c.1121A.G,p.D374G[[1](#_ENREF_1)]; c.1593A>T/p.K531N[[2](#_ENREF_2)]; c.92G>A/p.R31Q[[3](#_ENREF_3)]; c.1673G>A, p.G558D [[4](#_ENREF_4)] |
| STAT1 | c.800C>T/p.A267V, c.1074G>T/ p.L358F p.L358F/ c.863C>T/p.T288I[[5](#_ENREF_5)]; c.800C>T/p.A267V, c.863C>T/p.T288I, c.1074G>T/p.L358F, c.1170G>A, p.M390I[[6](#_ENREF_6)]; c.1154C>T/p.T385M, c.974T>A/p.M325K, c.862A>G/p.T288A, c.821G>A/p.R274Q, c.849G>T/p.L283F,  c.193G>A/p.D65N, c.1053G>T/p.L351F, c.2123C>T/p.S708F, c.988C>A/p.Q330K[[7](#_ENREF_7)] |
| *TNFSF5 (CD40L)* | Complex mutation in exon 5[[8](#_ENREF_8)]; g.IVS1-3T>G[[9](#_ENREF_9)]; g.IVS1+1G>A[[6](#_ENREF_6)]; IVS1-3T>G, IVS1+1G>A, IVS3+1G>A, IVS1-1 G>A, IVS4+1G>C, Large fragment deletion including exon4 and exon5[[10](#_ENREF_10)] |
| *IFNGR1* | c.182dupT/ p.V61fsX69[[6](#_ENREF_6)] |

[1] Lee PP, Chan KW, Lee TL, Ho MH, Chen XY, Li CH, Chu KM, Zeng HS and Lau YL. Penicilliosis in children without HIV infection--are they immunodeficient? Clin Infect Dis 2012; 54: e8-e19.

[2] Fan H, Huang L, Yang D, Lin Y, Lu G, Xie Y, Yu J and Zhang D. Pediatric hyperimmunoglobulin E syndrome: A case series of 4 children in China. Medicine (Baltimore) 2018; 97: e0215.

[3] Zhang W, Ye J, Qiu C, Wang L, Jin W, Jiang C, Xu L, Xu J, Li Y, Wang L and Jin H. Rapid and precise diagnosis of T. marneffei pulmonary infection in a HIV-negative patient with autosomal-dominant STAT3 mutation: a case report. Ther Adv Respir Dis 2020; 14: 1753466620929225.

[4] Pan M, Qiu Y, Zeng W, Tang S, Wei X and Zhang J. Disseminated Talaromyces marneffei infection presenting as multiple intestinal perforations and diffuse hepatic granulomatous inflammation in an infant with STAT3 mutation: a case report. BMC Infect Dis 2020; 20: 394.

[5] Lee PP, Mao H, Yang W, Chan KW, Ho MH, Lee TL, Chan JF, Woo PC, Tu W and Lau YL. Penicillium marneffei infection and impaired IFN-gamma immunity in humans with autosomal-dominant gain-of-phosphorylation STAT1 mutations. J Allergy Clin Immunol 2014; 133: 894-896 e895.

[6] Lee PP, Lao-Araya M, Yang J, Chan KW, Ma H, Pei LC, Kui L, Mao H, Yang W, Zhao X, Trakultivakorn M and Lau YL. Application of Flow Cytometry in the Diagnostics Pipeline of Primary Immunodeficiencies Underlying Disseminated Talaromyces marneffei Infection in HIV-Negative Children. Front Immunol 2019; 10: 2189.

[7] Chen X, Xu Q, Li X, Wang L, Yang L, Chen Z, Zeng T, Xue X, Xu T, Wang Y, Jia Y, Zhao Q, Wu J, Liang F, Tang X, Yang J, An Y and Zhao X. Molecular and Phenotypic Characterization of Nine Patients with STAT1 GOF Mutations in China. J Clin Immunol 2020; 40: 82-95.

[8] Kamchaisatian W, Kosalaraksa P, Benjaponpitak S, Hongeng S, Direkwattanachai C, Lumbiganon P and Wiangnon S. Penicillosis in Patients with X-linked Hyperimmunoglobulin M Syndrome (XHIGM), Case Reports from Thailand. J Allergy Clin Immunol 2006; 117:

[9] Liu D, Zhong LL, Li Y and Chen M. Recurrent fever, hepatosplenomegaly and eosinophilia in a boy. Zhongguo Dang Dai Er Ke Za Zhi 2016; 18: 1145-1149.

[10] Du X, Tang W, Chen X, Zeng T, Wang Y, Chen Z, Xu T, Zhou L, Tang X, An Y and Zhao X. Clinical, genetic and immunological characteristics of 40 Chinese patients with CD40 ligand deficiency. Scand J Immunol 2019; 90: e12798.
